# Quantitative Proteomic Analyses Identify STO/BBX24 -Related Proteins Induced by UV-B

**DOI:** 10.3390/ijms21072496

**Published:** 2020-04-03

**Authors:** Guizhen Lyu, Dongbing Li, Hui Xiong, Langtao Xiao, Jianhua Tong, Chanjuan Ning, Ping Wang, Shaoshan Li

**Affiliations:** 1 Key Laboratory of Ecology and Environmental Science in Guangdong Higher Education, School of Life Science, South China Normal University, Guangzhou 510631, Chinaldb06138233@163.com (D.L.); ncj1983@163.com (C.N.);; 2 Hunan Provincial Key Laboratory of Phytohormones, Hunan Agricultural University, Changsha 410128, Chinatjh0421@sohu.com (J.T.)

**Keywords:** UV-B irradiation, STO (salt tolerance)/BBX24, photosynthesis, antenna protein, flavanol, anthocyanin, NPQ (non-photochemical quenching)

## Abstract

Plants use solar radiation for photosynthesis and are inevitably exposed to UV-B. To adapt to UV-B radiation, plants have evolved a sophisticated strategy, but the mechanism is not well understood. We have previously reported that STO (salt tolerance)/BBX24 is a negative regulator of UV-B-induced photomorphogenesis. However, there is limited knowledge of the regulatory network of STO in UV-B signaling. Here, we report the identification of proteins differentially expressed in the wild type (WT) and *sto* mutant after UV-B radiation by iTRAQ (isobaric tags for relative and absolute quantitation)-based proteomic analysis to explore differential proteins that depend on STO and UV-B signaling. A total of 8212 proteins were successfully identified, 221 of them were STO-dependent proteins in UV-B irradiated plants. The abundances of STO-dependent PSB and LHC (light-harvesting complex) proteins in *sto* mutants decreased under UV-B radiation, suggesting that STO is necessary to maintain the normal accumulation of photosynthetic system complex under UV-B radiation to facilitate photosynthesis photon capture. The abundance of phenylalanine lyase-1 (PAL1), chalcone synthetase (CHS), and flavonoid synthetase (FLS) increased significantly after UV-B irradiation, suggesting that the accumulation of flavonoids do not require STO, but UV-B is needed. Under UV-B radiation, STO stabilizes the structure of antenna protein complex by maintaining the accumulation of PSBs and LHCs, thereby enhancing the non-photochemical quenching (NPQ) ability, releasing extra energy, protecting photosynthesis, and ultimately promoting the elongation of hypocotyl. The accumulation of flavonoid synthesis key proteins is independent of STO under UV-B radiation. Overall, our results provide a comprehensive regulatory network of STO in UV-B signaling.

## 1. Introduction

Plants grown in the natural environment are inevitably affected by UV-B. While animals can actively move to avoid UV radiation, plants have to adapt to UV-B radiation by synthesizing sunscreen substances such as flavonoids and anthocyanin [1,2]. High UV-B radiation induces stress responses in plants, such as damage to DNA, proteins, and the plasma membrane, accumulation of reactive oxygen species, cell cycle arrest, chlorophyll degradation, and inhibition of photosynthesis [3,4,5]. In recent years, UV-B has been revealed as a stress factor that alters gene expression related to physiological processes such as metabolism, morphogenesis, and photosynthesis [6]. UV-B also affects leaf development, circadian rhythm, and flowering time [7].

Previous biochemical assays identified UVR8 (UV resistance locus 8) as a UV-B receptor [8] and resolved its high-resolution structure [9]. UVR8 has a high sequence similarity with RCC1 (regulator of chromatin condensation 1) in humans and is highly conserved in plants [10]. In *Arabidopsis*, several protein complexes that receive and transmit UV-B signals have been investigated, including UVR8–COP1 (constitutively photomorphogenic 1), UVR8–WRKY36 (WRKY DNA-binding protein 36), and UVR8–BES1 (BRI1-EMS-suppressor1)/BIM1 (BES1-interacting MYC-like 1) [11]. The UV-B-induced transcription factor HY5 (elongated hypocotyl 5) is mediated by two major ways to activate downstream genes: (1) HY5 is synthesized in the presence of the nuclear UVR8-COP1 complex and then activates *HY5* [1,2,12]; (2) in the nucleus, the UVR8-WRKY36 complex prevents WRKY36 (a repressor of HY5) from binding to *HY5* promoter [13]. In addition, the UVR8-BIM1 complex dephosphorylates BES1, thus inhibiting the induction of BR-responsive genes related to hypocotyl elongation and plant growth by disrupting the formation of the BIM1-BES complex [14].

Our previous studies demonstrate the effect of salt tolerance (STO) on UV-B signaling. UV-B induces both the transcripts and protein levels of *STO*, and the *sto* mutants display defective hypocotyl elongation compared to the WT upon UV-B radiation. STO interacts with COP1 (an ubiquitin E3 ubiquitin ligase and a central mediator of UV-B signaling) in a UV-B-dependent manner. STO also suppresses HY5 transcription. In addition, STO interacts with RCD1 in vitro. Our genetic analysis reveal that the *rcd1-1* and *sto* mutant have similar hypocotyl length upon UV-B radiation, while 35S-STO-GFP overexpression could rescue the long hypocotyl phenotype of *rcd1-1* [15]. Moreover, STO is involved in other biological processes including salt stress [16], red and far-red light [17], circadian rhythm and flowering [3], and seedling development [18]. Hypocotyls of the *bbx24bbx25* mutant become shorter [19].

Recently, our research confirmed STO and GA negatively regulate UV-B-induced *Arabidopsis* root growth inhibition [20]. However, the mechanism by which STO regulates UV-B signaling is still unknown. Therefore, iTRAQ-based proteomics technology was used to investigate the mechanism of STO under UV-B radiation in the present research. Screening the STO-dependent UV-B-induced proteins lays a solid foundation for the later study of STO response to complex climate change. 

## 2. Results

### 2.1. General Characteristics of STO-Dependent UV-B-Induecd Proteins

To reveal the regulatory network of STO in UV-B signaling, we extracted total proteins from the *sto* mutant and WT (Col-0) for proteome analysis using iTRAQ. Ultimately, 8212 proteins were identified. We listed the detailed information of each protein in Table 1. 

**Figure 1:**
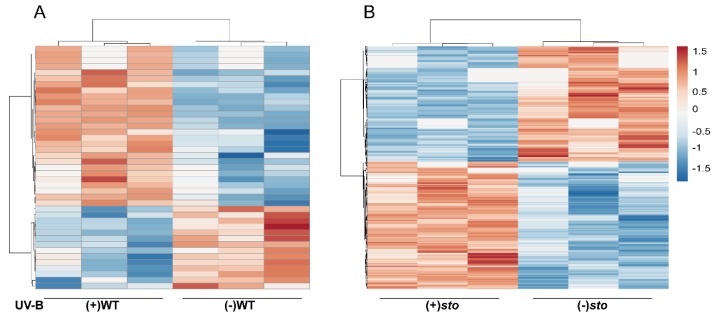
Clustering analysis of the differentially expressed proteins. (**A**) A hierarchical clustering analysis of the differentially expressed proteins in the wild-type (WT) seedlings with (+) or without (-) UV-B radiation. (**B**) A hierarchical clustering analysis of the differentially expressed proteins in the *sto* seedlings with (+) or without (-) UV-B. The bar represents the log_2_ fold change value.

Among the identified proteins, 41 differentially expressed proteins were from (+)WT/(-)WT, of which 14 were down-regulated regulated (fold change < 0.83, *P* < 0.05) and 27 were up-regulated (fold change > 1.2, *P* < 0.05). Three hundred and twenty-two differentially expressed proteins were from the UV-B-exposed *sto* mutant, of which 213 were up-regulated and 109 were down-regulated. A total of 614 differentially expressed proteins were from (+)*sto*/(+)WT, of which 292 were up-regulated and 322 were down-regulated (Table 2). Based on the abundance of the differentially expressed proteins (DEPs), a hierarchical clustering analysis was performed (Figure 1). The biological replicates showed good repeatability within each treatment group, but data between different treatment groups displayed low similarity. Moreover, according to the clustering analysis, the variation in protein abundance indicated the high frequency and complexity of differential protein expression in *sto* mutant after UV-B treatment (Figure 1B). The clustering analysis indicates that STO mediates a large portion of the differential protein expression in response to UV-B. 

**Figure 2:**
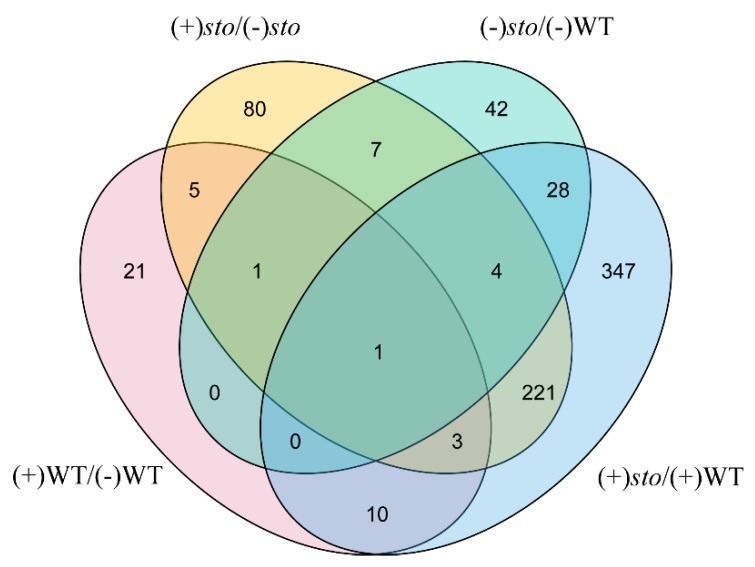
Venn diagram illustrating the number of identified proteins with abundance changed in WT and *sto* mutant before and after UV-B treatment.

As shown in Figure 2, we used TBtools [21] to draw a Venn diagram after comparison of DEPs between (+)WT/(-)WT and (+)*sto*/(+)WT groups. There were 221 STO-dependent UV-B response proteins and 39 UV-B dependent proteins in WT. 

**Figure 3:**
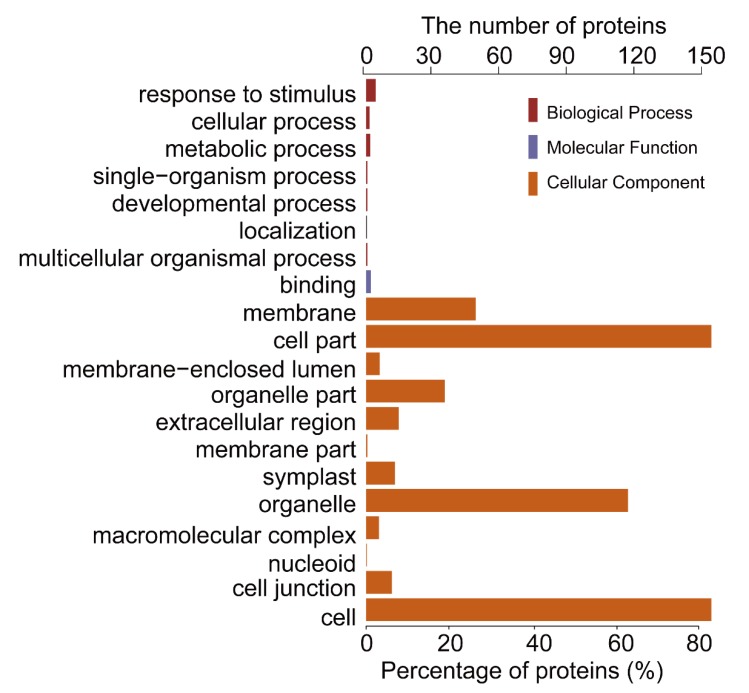
Gene ontology (GO) analysis of salt tolerance (STO)-dependent UV-B-induced proteins. GO analysis (gene ontology) describes the properties of genes and gene products in organisms from three aspects: the biological process (BP), the molecular function (MF), and the cell component (CC).

**Figure 4:**
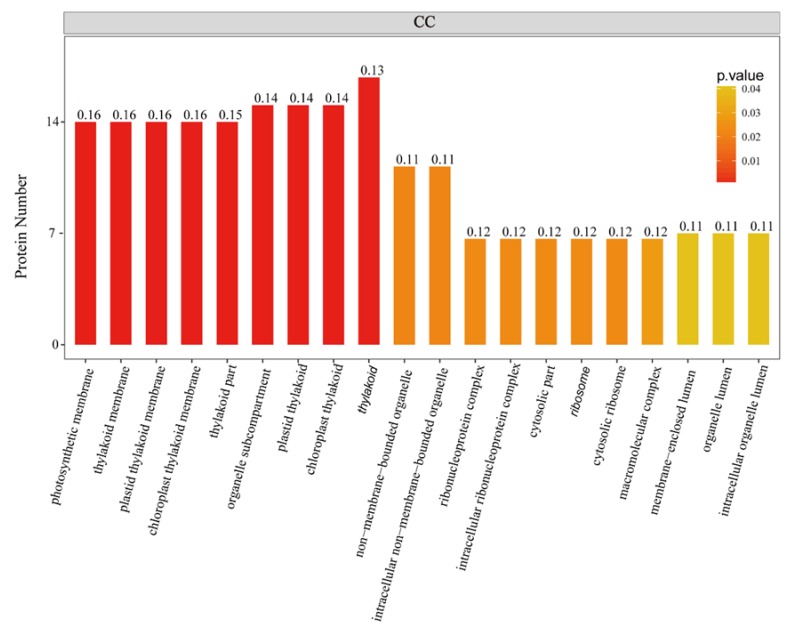
GO enrichment analysis of STO-dependent UV-B-induced proteins. The color gradient indicates the *P* value. The smaller *P* value indicates that the color is closer to red, the higher the level of GO enrichment. The number on the bar graph indicates the rich factor (rich factor <=1), referring to the percentage of differential proteins that are involved in this GO term.

To further investigate the biological function of the STO-dependent UV-B-induced proteins, we performed a GO (gene ontology) analysis. As shown in Figure 3, STO-dependent UV-B response proteins were distributed in the CC (cellular component) including cellular, membranes, organelles (chloroplasts, thylakoids, and mitochondria), mainly involved in UV-B light response stimulation, metabolism, plant development, and other important BP (biological processes), with the MF (molecular function) of activating transcription by binding promoters and combining with chlorophyll. Furthermore, we performed a GO enrichment analysis in CC. From the results of GO enrichment (Figure 4), it can be found that the UV-B response proteins dependent on STO were mainly located on the photosynthesis membrane, inner capsule membrane, chloroplast membrane, and thylakoid, indicating that STO is closely related to photosynthesis. 

**Figure 5:**
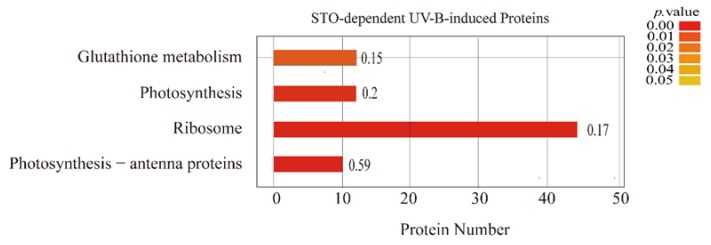
KEGG enrichment analysis of STO-dependent UV-B-induced proteins. The color gradient indicates the *P* value. The smaller *P* value indicates that the color is closer to red, the higher the level of pathway enrichment. The number on the bar graph indicates the rich factor (rich factor <=1), referring to the percentage of differential proteins that are involved in this pathway.

It is difficult for a single protein to perform its biological functions. The proteins in the organism need to coordinate with each other in an orderly way to perform their specific biological functions effectively. Therefore, we enriched those differential proteins that performed the same biological functions by the KEGG (Kyoto Encyclopedia of Genes and Genomes) pathway. The results are as follows: 59% of the proteins in the antenna protein complex and 20% of the proteins in photosynthesis changed significantly (Figure 5), which affected the light-harvesting capacity, photoelectron transfer, and photophosphorylation of photosynthesis. Therefore, the STO-dependent UV-B response proteins are most related to the assembly of antenna protein complex and photosynthesis pathway.

**Figure 6:**
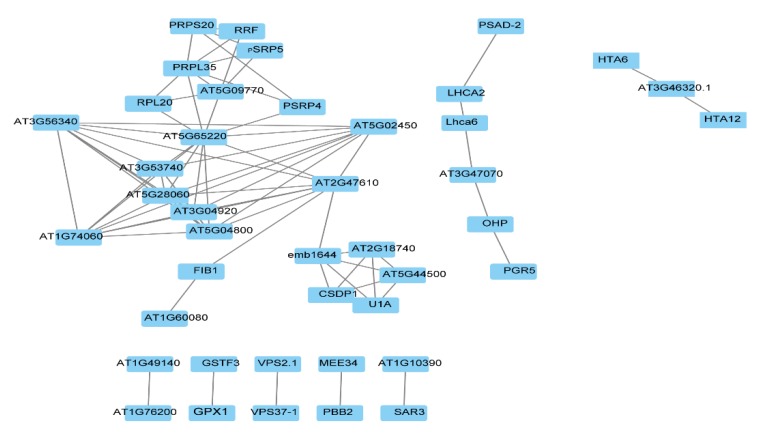
Protein interaction network (PPI) analysis of STO-dependent UV-B-induced proteins.

In order to find the key regulatory factors, the STRING database and Cytoscape software were used to construct the protein interaction network (PPI) to analyze the hub proteins in the network. The STO-dependent UV-B response proteins were filtered by Cytoscape, the top 20 proteins with the highest connectivity were selected for the map, and finally 8 PPI interaction networks were obtained (Figure 6). The function of interacting proteins is as follws: AT5G65220, as a hub protein, interacting with many proteins participates in cellular process; VPS2.1 (vacuolar protein sorting-associated protein 2 homolog 1) interacting with VPS37-1 participates in protein transport; SAR3 (suppressor of Auxin resistance 3) interacting with AT1G10390 participates in transport; AT1G76200 interacting with AT1G49140 participates in the oxidation–reduction process; GPX1 (glutathione peroxidase 1) interacting with GSTF3 (glutathione S-transferase F3) participates in the metabolic process; PBB2 (proteasome beta subunit PBB2) interacting with MEE34 (maternal effect embryo arrest 34) participates in the metabolic process; and PGR5 (proton gradient regulation 5) directly interacting with OHP (encodes a one-helix protein homologous to cyanobacterial high-light inducible proteins) and indirectly interacting with LHCA2 (Photosystem I light-harvesting complex gene 2) and PSAD-2 (Photosystem I reaction center subunit II-2) participates in photosynthesis and light reaction.

### 2.2. Changes of Candidate Proteins in Each Treatment Group

According to the results of enrichment analysis of GO, KEGG, and PPI protein interaction analysis, the proteins involved in antenna proteins and photosynthesis were listed. At the same time, considering the possible technical problems, the high-abundance proteins of plants were not removed in advance, and some low-abundance proteins (such as transcription factors) were not identified, leading to the failure of enrichment of some pathways. The proteins of flavonoid synthesis and metabolism and hormone signal transduction pathways were also listed for analysis. UV-B radiation promotes the accumulation of flavonoids and anthocyanins, changes hormone synthesis and signal regulation, and activates the anti-stress mechanism in plants. NS in the table indicates that the difference is not significant (*P* < 0.05), and the ratio is the multiple of protein expression difference (ratio > 1.2, indicating up-regulated; ratio < 0.83, indicating down-regulated). After summarizing the changes of different proteins in different treatment groups, it is speculated that the STO protein not only responds to UV-B, but also is closely related to stress response. The specific protein change information is as follows.

(1) Changes of differential protein expression related to flavonoids synthesis and metabolism. The expression of differential proteins from (+)WT/(-) WT and (+)*sto*/(-)*sto* including phenylalanine lyase1/2 (PAL1/2), chalcone synthetase (CHS), flavonoid synthetase (FLS), and UDP glycosyltransferase were higher than that without UV-B radiation. However, the key enzymes of the flavonoid pathway in *sto* mutants were also significantly up-regulated after UV-B radiation. Compared with the WT, the *sto* mutant after UV-B radiation showed no significant difference. The results show that UV-B notably induces and accumulates flavonoids in plants to adapt to the environment. The accumulation of flavonoids do not depend on STO, and there are other proteins to regulate the expression of flavonoids (Table 3).

(2) Changes of differential protein expression involved in photosynthesis. As shown in Table 4, there was no significant change in the expression of photosynthesis-related proteins in the WT before and after UV-B radiation. Similarly, there was no significant change in the expression of photosynthesis-related proteins in sto mutant and WT control groups without UV-B radiation. In the comparison group of (+)*sto*/(-)*sto* and (+)*sto*/(+)WT, the protein expressions of PSBs and LHCs decreased significantly, which indicates that the absence of STO under UV-B radiation would affect photosynthesis.

(3) Changes of differential protein expression involved in hormones. In Table 5, the negative regulatory transcription factor ERD15 (early response to dehydration 15) of ABA (abscisic acid) signal significantly decreased 0.78 times in (+)WT/(-)WT, 1.6 times in (+)*sto*/(-)*sto*, and 1.9 times in (+)*sto*/(+)WT, indicating that ERD15 may respond to UV-B radiation. STO may negatively regulate the expression of ERD15 in UV-B signal transduction. The proteins P5CS1 (delta-1-pyrroline-5-carboxylate synthase 1), RD22 (BURP domain protein RD22), and LTP3 (lipid-transfer protein 3) related to ABA signal transduction were significantly down-regulated in (+)*sto*/(+)WT, indicating that STO may promote the expression of proteins related to ABA signal transduction, but not relate to UV-B radiation. Lox2 (lipoxygenase 2) related to JA (jasmonate) was significantly down-regulated in (-)*sto*/(-)WT and (+)*sto*/(+)WT, indicating that STO promotes Lox2 protein expression and communicated with JA signal, but does not depend on UV-B radiation. DRM2 (DNA (cytosine-5)-methyltransferase) related to IAA (auxin) and methylation was down-regulated in (+)WT/(-)WT and up-regulated in (+)*sto*/(+)WT, suggesting that STO may inhibit the expression of DRM2 in UV-B signal transduction and reduce the degree of methylation in plants. Auxin efflux carrier family protein and AXR4 (auxin response 4) were significantly up-regulated in (+)*sto*/(-)*sto* and (+)*sto*/(+)WT, indicating that STO may inhibit the expression of auxin efflux carrier family protein and AXR4 under UV-B radiation, and communicate with IAA signals. ACO2 (aconitate hydratase 2)-related ethylene was significantly up-regulated in (+)*sto*/(-)*sto* and (+)*sto*/(+)WT, indicating that STO inhibits the expression of ACO2, but does not depend on UV-B radiation.

### 2.3. Verification of Differential Protein

**Figure 7:**
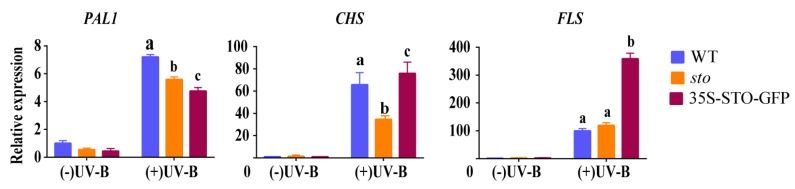
Expression profile of genes involved in flavonoid synthesis before and after UV-B treatment. The WT, *sto,* and 35S-STO-GFP seedlings were grown in white light for seven days and then subjected to a four-hour UV-B treatment (0.6 W/m^2^). Error bars indicate the SEM of three independent biological and technical replicates. Different letters indicate significant differences between gene relative expressions of those seedlings grown under UV-B radiation (*P* < 0.05, Dunnett’s multiple). *Actin2* was used as an internal reference gene.

**Figure 8:**
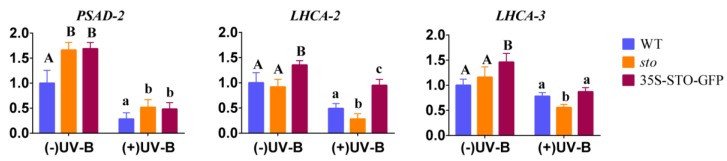
Expression profile of photosynthesis genes before and after UV-B treatment. The WT, *sto,* and 35S-STO-GFP seedlings were grown in white light for seven days and then subjected to a four-hour UV-B treatment (0.6 W/m^2^). Error bars indicate the SEM of three independent biological and technical replicates. Different capital letters and lowercase letters indicate significant differences between gene relative expression of those seedlings grown without or with UV-B radiation, respectively (*P* < 0.05, Dunnett’s multiple). *Actin2* was used as an internal reference gene.

**Figure 9:**
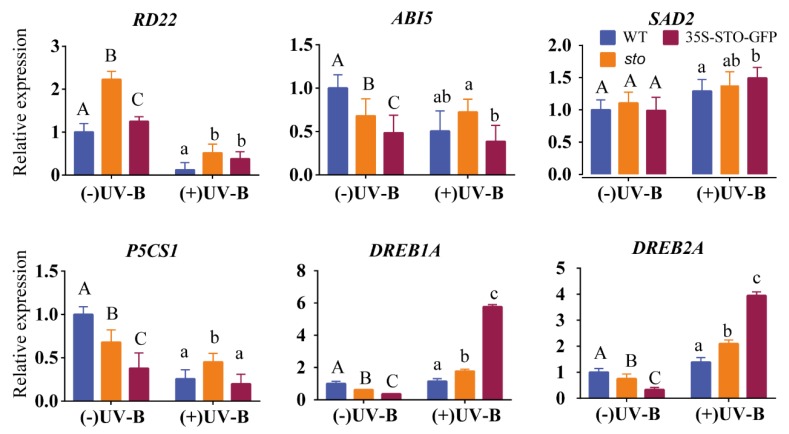
Expression profile of ABA pathway genes before and after UV-B treatment. The WT, *sto,* and 35S-STO-GFP seedlings were grown in white light for seven days and then subjected to a four-hour UV-B treatment (0.6 W/m^2^). Error bars indicate the SEM of three independent biological and technical replicates. Different capital letters and lowercase letters indicate significant differences between gene relative expression of those seedlings grown without or with UV-B radiation, respectively (*P* < 0.05, Dunnett’s multiple). *Actin2* was used as an internal reference gene.

(1) qRT-PCR verification of differential genes related to flavonoid synthesis and metabolism. The results of proteomics show that UV-B significantly induces the accumulation of flavonoids in plants, and the accumulation of flavonoids do not depend on STO (Table 3). Therefore, the transcriptional levels of *PAL1*, *CHS*, and *FLS* in the flavonoid biosynthesis pathways of WT, *sto* mutant, and 35S-STO-GFP plants were detected after UV-B radiation for 4 h. As shown in Figure 7, *PAL1* in WT, *sto* mutant, and 35S-STO-GFP plants were up-regulated by more than 5 times after UV-B radiation, but the expression levels of *PAL1* in *sto* mutant and 35S-STO-GFP plants were significantly lower than that in WT, indicating that STO does not regulate the transcription level of *PAL1* in UV-B signaling. The *CHS* in WT, *sto* mutant, and STO overexpressing plants increased by more than 40 times after UV-B radiation, but the expression level of *CHS* in *sto* mutant was significantly lower than that in WT and STO overexpressing plants, indicating that STO may regulate the transcription level of *CHS* in the UV-B pathway. FLS in WT, *sto* mutant, and 35S-STO-GFP plants were up-regulated by more than 100 times after UV-B radiation, but *FLS* expression level in 35S-STO-GFP plants was significantly lower than that in WT and *sto* mutant, and there was no significant difference in FLS transcription levels between WT and *sto* mutant. It is suggested that the up-regulated expression of *FLS* after UV-B radiation does not depend on STO, but STO overexpression can promote FLS transcription and flavonoid accumulation.

(2) qRT-PCR verification of differential genes involved in photosynthesis. The results of proteomics show that STO relates to the antenna protein (Table 4). The transcription levels of *PSAD-2*, *LHCA2*, and *LHCA3* (Photosystem I light harvesting complex gene 3) in WT, *sto* mutant, and 35S-STO-GFP plants were measured after 4 h UV-B radiation. As shown in Figure 8, the gene expression levels of *PSAD-2*, *LHCA2*, and *LHCA3* in 35S-STO-GFP lines were significantly higher than those in WT and *sto* mutant without UV-B radiation, indicating that STO can improve the photosynthesis of plants. After UV-B radiation, *PSAD-2*, *LHCA2*, and *LHCA3* were significantly down regulated in WT, *sto* mutants, and 35S-STO-GFP plants after UV-B radiation, indicating that UV-B radiation affects photosynthesis of plants. After UV-B radiation, the *PSAD-2* gene expression level of STO overexpressed plants was higher than that of WT, the *LHCA2* gene expression level was higher than that of WT and *sto* mutant, and the *LHCA3* gene expression level was higher than that of *sto* mutant. The results indicate that STO could restore the photosynthetic capacity after UV-B radiation and reduce the light damage caused by UV-B.

(3) qRT-PCR verification of differential genes involved in hormones. To gain further insight into the crosstalk between UV-B and the ABA pathway, we measured the transcript levels of *RD22*, *AB15* (ABA insensitive 5), *P5CS1*, *SAD2*, *DREB1A* (dehydration response element B1A, encodes a member of the DREB subfamily A-1 of ERF/AP2 transcription factor family (CBF3)), and *DREB2A* (dehydration response element B2A) under UV-B radiation (Figure 9). *RD22*, *AB15*, and *P5CS1* were down-regulated in the WT and 35S-STO-GFP seedlings but were up-regulated in *sto* mutant, indicating the inhibition of *RD22*, *AB15*, and *P5CS1* transcription by STO under UV-B radiation. The transcript levels of SAD2 do not have a significant difference between all the genotypes, indicating that UV-B does not affect *SAD2* transcription. *DREB1A* and *DREB2A* were significantly up-regulated after UV-B treatment, indicating that these two genes activate by STO in a UV-B-dependent manner (Figure 9). These results provide evidence for a crosstalk between STO and ABA signaling.

(4) PRM (parallel reaction monitoring) verification of differential proteins. To validate the iTRAQ data, we selected five proteins within the potentially relevant pathways (anthocyanin biosynthesis, photosynthesis, and protein synthesis) for PRM quantification (Table 6). The PRM results demonstrated the consistency in the differential expression patterns of selected proteins with the iTRAQ data. LHCB6, PSAD-2, and RPL5B were up-regulated in (+)WT/(-)WT and down-regulated in (+)*sto*/(+)WT. In (+)WT/(-)WT, the expression levels of PAL1 and UGT84A2, which are involved in anthocyanin biosynthesis, show a significant increase in both the PRM and iTRAQ analyses. In (+)*sto*/(+)WT, PAL1 and UGT84A2 were up-regulated in the PRM data and were unchanged according to the iTRAQ results. In general, consistency between the PRM and the iTRAQ results validated the credibility of iTRAQ in proteomic analyses.

### 2.4. STO Regulates Hypocotyl Elongation and Protects the Photosynthetic System after UV-B Treatment

**Figure 10:**
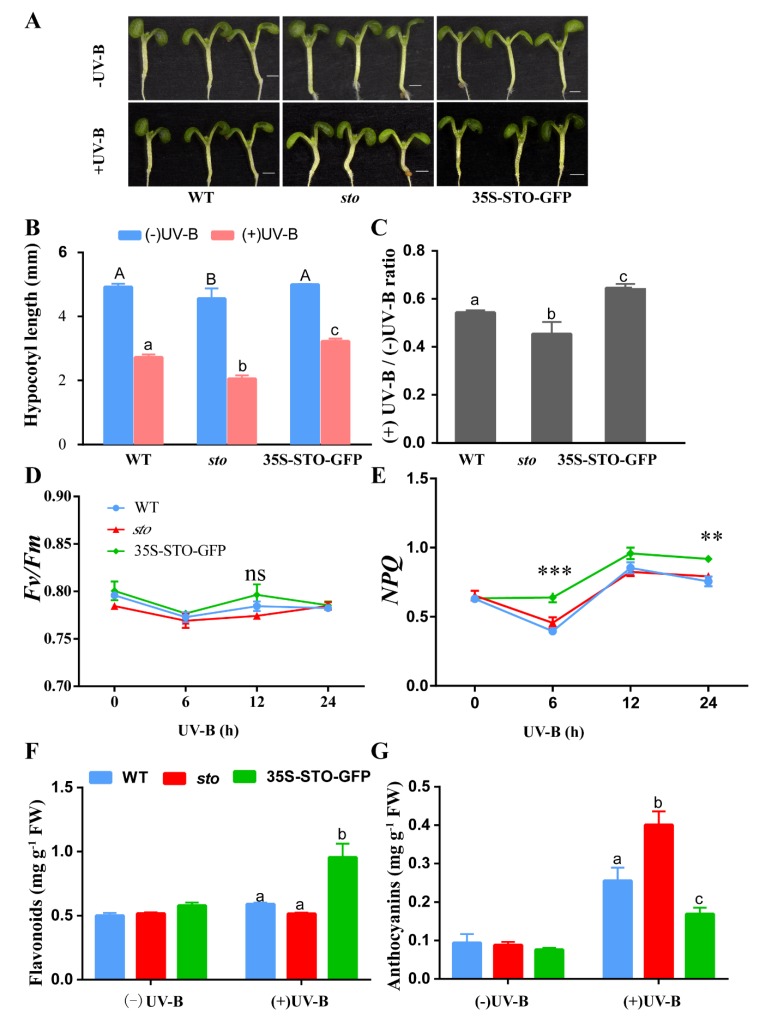
Physiological responses of STO under UV-B radiation. (**A**) Images of the six-day-old *sto*, WT, and 35S-STO-GFP seedlings with or without UV-B radiation. UV-B radiation was 0.6 W/ m^2^; scale bar = 1 mm. (**B**) Hypocotyl length of the *sto*, WT, and 35S-STO-GFP seedlings with or without UV-B radiation. Data are expressed as means ± standard deviation (SD); *n* > 30. The upper- and lower-case letters indicate the results of the Dunnett’s multiple comparisons test on the difference in hypocotyl length between those seedlings without or with UV-B exposure, respectively (*P* < 0.05). (**C**) The grey bars show the ratio of hypocotyl length with or without UV-B radiation (+UV-B/-UV-B). Data are shown as means ± SD (*n* > 30). Different letters indicate statistically significant differences (*P* < 0.05, one-way ANOVA). (**D**) and (**E)** Fv/Fm and NPQ of *sto*, WT, and 35S-STO-GFP seedlings after UV-B exposure. The seedlings were grown in white light for seven days and then exposed to UV-B (0.6 W/ m^2^). Fv/Fm and NPQ were measured using CF Imager (Technologica). Data are shown as mean ± SD (*n* > 20). “*” indicates a statistically significant difference, and “ns” means no significant difference (*P* < 0.05, Dunnett’s multiple comparisons test). (**F**) and (**G**) Anthocyanin and flavonoid contents in the seedlings with or without UV-B radiation. The seedlings were grown in the soil under white light for 12 d and then subjected to a three-day UV-B (0.6 W/m^2^) exposure. The seedlings were then measured for anthocyanin and flavonoid contents. Data are shown as means ± SEM (*n* = 6). Different letters indicate significant difference (*P* < 0.05, Dunnett’s multiple comparisons test).

In the absence of UV-B radiation the hypocotyls of the *sto* mutant were shorter than those of the WT and 35S-STO-GFP seedlings. However, the inhibitory effect of UV-B radiation on hypocotyl growth was compromised in 35S-STO-GFP. The *sto* mutants were more sensitive to UV-B (the hypocotyl length ratio of + UVB/-UV-B was 0.45 for the *sto* mutant, 0.54 for the WT, and 0.64 for 35S-STO-GFP). The hypocotyls of the 35S-STO-GFP seedlings were longer than those of the WT and *sto* mutant seedlings after UV-B treatment (Figure 10A–C). The values of *Fv/Fm* in WT, 35S-STO-GFP, and the *sto* mutant showed no obvious changes and ranged from 077 to 0.79 within 24 h after UV-B radiation, but NPQ in the 35S-STO-GFP seedlings was much higher than that of WT and the *sto* mutant. NPQ peaked at 12 h after UV-B radiation in all three genotypes (Figure 10D,E). After the UV-B treatment, the anthocyanin (UV-absorbing compounds) content in the *sto* mutant increased by 4-fold, while the level of flavonoid remained the same as the *sto* mutant. A 2-fold increase in flavonoid level was observed in 35S-STO-GFP, and the anthocyanin level was doubled upon UV-B radiation. The level of anthocyanin increased by 2-fold in the WT, whereas flavonoid content was only slightly elevated under UV-B treatment. According to the results above, UV-B exposure accelerated anthocyanin accumulation in the *sto* mutant (Figure 10F,G). Taken together, these phenotypes demonstrate that STO positively regulates hypocotyl elongation by antagonizing UV-B repression. These results also indicate flavonoids and STO play a significant role in plant photoprotection upon UV-B exposure.

### 2.5. STO Affects the Content of ABA, JA, and IAA upon UV-B Exposure

**Figure 11:**
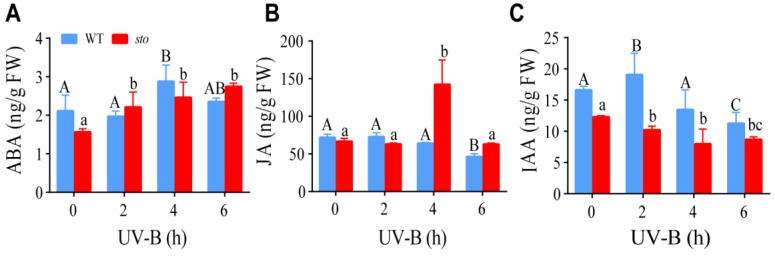
Changes of endogenous hormones ABA, JA, and IAA in wild-type and *sto* mutant under UV-B radiation. The seedlings of WT and *sto* were treated with UV-B (0.6 W/m^2^) for 2, 4, and 6 h, respectively. The seedlings in the soil grew under white light for 12 d, then irradiated with UV-B (0.6 W/m^2^). Error bars indicate the SEM of three independent biological and technical replicates. Different capital letters and lowercase letters indicate significant differences between changes of endogenous hormones of those seedlings grown without or with UV-B radiation, respectively (*P* < 0.05, Dunnett’s multiple).

The promoters of STO have response elements of IAA, ABA, and JA [22]. Our proteomics data indicate STO has a crosstalk with ABA, JA, and IAA signaling. Therefore, we measured the contents of these hormones in WT and the *sto* mutant upon UV-B exposure. LC-MS/MS analysis revealed an increase in ABA level in the WT and *sto* mutant after UV-B radiation. JA content in the WT slightly decreased after UV-B radiation. Interestingly, a sharp increase in JA was observed in the *sto* mutant 4 h after UV-B radiation, followed by a decrease to the normal level at 6 h. IAA level in the WT and the *sto* mutant decreased after UV-B radiation (Figure 11). These results suggest that UV-B may inhibit hypocotyl elongation by inhibiting IAA synthesis or promoting ABA synthesis.

## 3. Discussion

Many studies have reported the UV-B signaling pathway in *Arabidopsis*. UVR8 is a major photoreceptor [11], and the COP1–SPA (suppressor of PHYA-105) complex and many other key factors are also involved in UV-B photomorphogenesis, e.g., CUL4-DDB1 (cullin 4-DNA damage binding protein 1) [2,23], STO [15], BES1 and BIM1 [14], WRKY36 [13], HY5 [12], and RUP1 (repressor of UV-B photomorphogenesis 1) and RUP2 (repressor of UV-B photomorphogenesis 2) [24]. However, the orchestration of the downstream UV-B signaling pathway by STO is still poorly understood. Our study explores the regulatory network of STO using the iTRAQ method in *Arabidopsis* and demonstrates that STO could interfere with anthocyanin biosynthesis, promote the accumulation of flavonoid biosynthesis and antenna proteins, and crosstalk with the ABA pathway upon UV-B radiation.

### 3.1. The Accumulation of Flavonoids and Anthocyanins under UV-B Radiation is Independent of STO

As a small part of the solar spectrum, UV-B radiation has a significant impact on plant secondary metabolism, especially the accumulation of flavonoids (flavonoids and anthocyanins), which is considered to be one of the most important protective reactions to UV-B radiation. Flavonoids can filter UV-B before the UV-B signal is transmitted to sensitive molecules in mesophyll cells and causes oxidative degradation of membrane lipids [25,26,27]. However, UV-B damages DNA, proteins, and membranes, and it hinders photosynthesis and plant growth [28]. Our results show that UV-B increases the transcription (Figure 7) and protein levels (Table 3) of key enzymes in flavonoid synthesis, but their expressions do not depend on STO.

UV-B has a minor direct effect on photosynthesis but induces the accumulation of UV-absorbing flavonoids and anthocyanin under UV-B (Figure 10). In many plant species, UV-B up-regulates the expression of the phenylpropanoid and flavonoid biosynthetic genes [29]. Flavonoids and anthocyanin function as sunscreen substances [30]. Several biosynthetic enzymes, such as PAL, CHS, FLS, and UGT84A2, are up-regulated under UV-B light (Table 3). PAL initiates the synthesis of anthocyanin and other flavonoids, whereas CHS produces tetrahydroxy-chalcone [31]. FLS converts dihydroflavonol to flavonoids, whereas UFGT is an anthocyanin synthase that stabilizes anthocyanin [32]. Several flavonol and anthocyanin biosynthetic genes can be activated by HY5 and R2R3-MYBs [33]. However, a small amount of anthocyanin could still be detected in the tomato *hy5* mutant, indicating the existence of some HY5-independent transcription factors that mediate anthocyanin metabolism [34]. 

The flavonoid content was increased, and the anthocyanin content was decreased in the 35S-STO-GFP seedlings after UV-B induction (Figure 10). UGT84A2, a key anthocyanin biosynthetic enzyme, was up-regulated in the *sto* mutant (Table 6). The E3 ligase CUL4-DDB1–RUP1/RUP2 complex mediates HY5 degradation under UV-B radiation, thus inhibiting UV-B–induced anthocyanin accumulation. The transcript levels of *CHS, UGT84A, ELIP1,* and *ELIP2* were up-regulated in double-mutant *rup1-1 rup2-1* seedlings. Meanwhile, COP1 directly targets RUP1/RUP2 for degradation, which alleviates RUP1/RUP2 accumulation and COP1–HY5, thus stabilizing HY5 under UV-B radiation [35]. The accumulation of anthocyanin in three *myb* mutants was higher than that in *hy5* mutants, indicating that the accumulation of anthocyanin must require the accumulation of HY5 [36]. In *Arabidopsis*, flavonoid metabolism is precisely controlled. For example, miR156-mediated transcript cleavage reduces anthocyanin accumulation and enhances flavonoid accumulation [37]. MYB, bHLH, and WD40 are the main transcription factors that regulate anthocyanin synthesis, whose protein complexes (MBW) are bound to the promoters of structural genes for regulation [38,39,40]. COP1 and SPA (suppressor of phya-105) inhibit photoinduced anthocyanin biosynthesis by targeting the key active factors (PAP1, PAP2, HY5, and their homologues, HYH) in anthocyanin synthesis in *Arabidopsis* [41]. mir828/TAS4-siR81(-) negatively regulates anthocyanin accumulation by inhibiting the expression of positive regulatory factors PAP1, PAP2, and MYB113 [42].

How STO regulates the balance between anthocyanin and flavonoids is still unknown. STO may affect proteins related to the synthesis of flavonoids by regulating the transcription of HY5. However, the expression of HY5 protein was not identified in the differential proteomic data, which may be due to the low expression of transcription factors covered by high-abundance proteins, resulting in many reported transcription factors not identified.

### 3.2. STO Promotes Photosynthesis-Antenna Protein Accumulation

Photosynthesis is the most important chemical reaction on the earth, which can provide energy and material sources for life activities. Photosystem II (PSII) and photosystem I (PSI) promote the absorption, transmission, and transformation of light energy in photosynthesis. In the face of adversity, plants reduce energy supply and resist various abiotic stresses by inhibiting photosynthetic and energy release reaction. Photosynthetic electron transport and many metabolic reactions are carried out in chloroplasts, and environmental stress easily affects the metabolism balance of chloroplasts. PSI-LHCI in green algae is combined with more antenna protein complexes, indicating that an increase in the ratio of pigment/reaction center of antenna protein complexes is conducive to the PSI capturing more photons [43]. With the enhancement of UV-B radiation, the ability of electron transfer in plants decreases, the content of Cytf decreases, PSI protein damages, and ATPase and photophosphorylation activity decreases [44,45,46,47].

To protect the photosynthetic machinery, UV-B significantly induces the quantum efficiency (qE) capacity in *Chlamydomonas reinhardtii*, as several key contributors to qE, including LHC Stress-Related 1 (LHCSR1) and PSBS, are up-regulated by UVR8 [48]. NPQ also participates in the regulation of light harvesting, dissipating excess energy as heat through the major and most rapid component, qE (energy-dependent component), to circumvent photodamage [49]. PsbS belongs to the LHC protein superfamily [50] and is essential for qE [51]. In *Arabidopsis*, PSB29/THF1 is important for the normal accumulation of the FtsH heterocomplex involved in PSII repair [52]. The antenna system is composed of proteins of the LHC family and antenna complexes that protect photosystem I from photoinhibition [53]. NPQ also requires lipocalin proteins [54,55] such as V de-epoxidase (VDE) and the thylakoid membrane stromal-located zeaxanthin epoxidase (ZEP) [56]. Therefore, interference with NPQ may be caused by the down-regulation of lipid transporter proteins in the thylakoid. 

Through comparative proteomic analysis, it has been found that the expression abundances of some components of PSI (PsaF) and PSII (D1, LHCA, LHCB) detected in *sto* mutant decreased (Table 4). The results show that STO is necessary for the normal accumulation of photopigment protein complex. It is speculated that the rate of photosynthetic electrons in *sto* mutant might be decreased. It may be related to the need for plants to face high-energy UV-B light environment changes, forcing plants to sacrifice energy transfer rate in growth to cope with high light intensity stress for nonphotochemical quenching. High-energy UV-B radiation damages PSII and PSI, affects photosynthesis, and then affects life activities. The results of proteomics suggest that STO could maintain the normal structure of antenna protein complex under UV-B radiation, improve the photosynthetic electron transfer rate to meet the needs of survival, and adapt to UV-B radiation. Overexpression of STO under UV-B radiation significantly increases the NPQ level by detecting chlorophyll fluorescence parameters (Figure 10). STO maintains the accumulation of LHC family proteins under UV-B radiation and improves the heat dissipation ability of NPQ through comparative proteomic analysis (Figure 10). The UV-B response proteins dependent on STO are mainly located on thylakoid membrane and chloroplast membrane (Figure 4 and Figure 5). Many LHC family proteins in *sto* mutant were down regulated after UV-B radiation (Table 4), and the light protection ability decreased. On the contrary, the NPQ ability of 35S-STO-GFP seedlings was enhanced, which could release the extra energy of UV-B and reduce the damage (Figure 10). Therefore, the interference of UV-B on NPQ (Figure 11) may be caused by the down regulation of LHC family protein. STO could stabilize the normal structure of light harvesting protein complex, perform photosynthesis, and maintain the normal metabolism of life activities by maintaining the accumulation of antenna protein complex, improving the NPQ ability, and releasing extra energy.

### 3.3. STO Had Crosstalk with ABA upon UV-B Radiation

The plant hormone ABA inhibits root elongation and seed germination. According to the proteomics data (Table 5), auxin and ABA signaling factors strongly associate with STO in a UV-B-dependent manner. In the *sto* mutant, the expression of auxin-related proteins (DRM2, AXR4) and negative regulatory protein ERD15 of ABA were up-regulated after UV-B radiation. The *axr1-3* and *ibr5-1* mutants grown on MS plates containing 10 μM ABA had a similar root length, which was greater than that of the WT, suggesting that AXR1 promotes ABA signaling and inhibits root elongation [57]. We found that *AXR4* and *ABI5* were significantly up-regulated in the *sto* mutant under UV-B radiation (Table 5 and Figure 9), suggesting STO may attenuate ABA signaling and promote hypocotyl elongation by inhibiting AXR4 expression. 

The accumulation and distribution of auxin in *drm1-drm2-cmt3* mutants were affected [58]. DNA methylation is an epigenetic modification of DNA, which plays an important role in the development of embryo, organ, flower and fruit, and the response of plants to different stresses [59]. Mutations in DNA methyltransferase lead to DNA methylation loss, affect gene expression regulating auxin synthesis, transport and signaling pathways, and lead to developmental abnormalities [58]. DNA methyltransferase CMT3 and DRM2 were up-regulated to increase glutathione reductase (GR), ascorbic acid peroxidase (APX), and catalase (CAT) under Cu or Cd stress, which improve resistance to heavy metals in plants [60]. DRM2 protein expression was significantly up-regulated in (+)*sto*/(+)WT and down-regulated in (+)*sto*/(+)WT (Table 5), suggesting that the DNA methylation degree of plants increases with STO mutation after UV-B radiation, which may maintain survival by sacrificing growth energy and improving plant resistance.

Transcription factors DREB1A/CBF3 and DREB2A interact specifically with cis-acting elements (DRE, CRT) related to cold and drought stress response gene expression in *Arabidopsis*. Overexpression of constitutive activity *DREB2A* enhances drought tolerance and regulates the expression of many water stress-induced genes [61]. *DREB1A/2A* in 35S-STO-GFP plants were significantly up-regulated after UV-B treatment (Figure 9).

The ERD (Early Responsive to Dehydration) genes can be rapidly induced upon abiotic stresses such as drought, low temperature, or salinity [62]. Belonging to a highly conserved family, ERD15 is not only a central component of several stress responses in *Arabidopsis* but also involved in stomatal closure. Previous studies have reported the sequence similarity between the light stress-regulated genes (*Lsr1*) and *AtERD15*, as well as the significant *Lsr1* transcription inhibition by UV-A irradiation [63]. Now, the molecular mechanism of ERD15 in UV-B signaling is not known. Our results suggest that STO may repress ERD15 to enhance ABA signaling. 

## 4. Materials and Methods 

### 4.1. Plant Materials and UV-B Treatment

The seedlings of *Arabidopsis thaliana* wild-type Columbia (Col-0), *sto* mutant, and 35S::STO-GFP [15] were used. After surface sterilization in 30 % (*v/v*) chlorine bleach (sodium hypochlorite) for 13 min, the seeds were sown on 1/2 MS (half-strength Murashige and Skoog) medium containing 3% sucrose and 0.8% agar. The seeds went through a four-day stratification at 4 °C in the dark before grown in white light (100 µmol m^−2^ s^−1^) for seven days (light/dark, 16/8 h) at 21 °C. We performed the UV-B treatment in narrowband UV-B tubes (Philips TL 20W/01 RS) at 0.6 W m^–2^ s^−1^, with a radiation rate at approximately 0.785 µmol m^−2^ s^−1^. After UV-B radiation, the seedlings were grown under 10 µmol m^−2^ s^−1^ white light for 4 h. To filter UV-B and block UV-C, we used the cellulose acetate film (+UV-B). To block both UV-B and UV-C, we used the mylar film (-UV-B). The Quantithern Light Meter (Hansatec) was used to measure light intensity. 

### 4.2. Protein Extraction and Digestion

For protein lysis, 300 mg randomly selected samples from WT and *sto* mutant seedlings exposed to UV-B were ground with liquid nitrogen and homogenized in SDT (4% SDS, 100 mM Tris-HCl, 1 mM DTT, pH7.6) buffer. The BCA Protein Assay Kit (Bio-Rad) was used to quantify the proteins. Trypsin was used to digest the proteins into peptides according to Matthias Mann [64]. After digestion, the samples went through desalting using the C18 Cartridges (standard density, bed I.D. 7 mm, 3 mL, Sigma) and were concentrated by vacuum centrifugation and reconstitution in 40 µL dissolution buffer. A280 was measured to quantify the peptides.

### 4.3. iTRAQ Labeling, Strong Cation Exchange (SCX) Fractionation, and LC-MS/MS Analysis

A 100 μg peptide mixture of each sample was labeled with the iTRAQ reagent according to the manufacturer’s instructions (Applied Biosystems). The labeled samples were designated (-)UV-B-Col/114, (+)UV-B-Col/115, (-)UV-B-*sto*/116, and (+)UV-B-*sto*/117 (sample/isobaric tag). We prepared three replicates for each sample. We used SCX chromatography to fractionate each labeled sample into 15 parts before desalting and lyophilization [65]. The resulting fractionated samples were separated on an Easy nLC (nanoliter flow rate) HPLC system (Thermo Scientific). The samples were chromatographed and subjected to 1 h gradient Q-Exactive analysis 45 times as described previously [65].

### 4.4. Protein Characterization

After obtaining the MS/MS data, we used MASCOT v2.3.02 (Matrix Science, Berkshire, UK) to identify and quantify the proteins using Proteome Discoverer 1.4. Before the protein search, we set the enzyme as trypsin, MS/MS fragment ion mass tolerance at ±0.1 Da, peptide mass tolerance at ±20 ppm, missed cleavages at 2, fixed modifications as carbamidomethylation (Cys) and iTRAQ 4plex (N-terminal and Lys), and variable modifications as oxidation (Met) and iTRAQ 4plex (Tyr). The TAIR10_pep_20101214.fasta database was used. The maximum false discovery rate (FDR) of the data was 5% to be eligible for quantification. For protein ratio calculation, we employed the median of unique peptides, normalizing all peptide ratios by the median (normalized to 1 prior to normalization of all peptide ratios). We also verified the repeatability of the replicates by comparing the protein abundance of each biological replicate to 1. Proteins with an expression level change of >1.2 (up-regulated) or < 0.83 (down-regulated) were considered DEPs; *P*-value <0.05.

### 4.5. Function Annotation

The proteomics data were further processed using Clustvis (https://biit.cs.ut.ee/clustvis/). The DEPs were functionally annotated using Blast2GO [66] and were mapped for KEGG pathways. The protein ID number of the target protein was converted to the KO (KEGG Orthology) number through Uniprot website. KEGG paththway annotation was completed through the KEGG online website (https://www.kegg.jp/) [67]. The distribution of the KEGG pathway in the target protein set and identified protein set was analyzed by Fisher’s exact test. The interaction between the target proteins can be analyzed through the STRING (http://string-db.org/) database. The interacting network was generated by CytoScape software (version number: 3.2.1, http://www.cytoscape.org/).

### 4.6. Quantitative Real-Time PCR

Total RNA was extracted using RNAiso Plus (#9108, TaKaRa, Dalian, China), and cDNA was synthesized using PrimeScript™ RT reagent Kit with gDNA Eraser (#RR047A, TaKaRa, Dalian, China) for qRT-PCR reactions on the QuantStudio 6 Flex Real-Time PCR System (Applied Biosystems, Foster City, USA). We used Luna® Universal qPCR Master Mix (#M3003L, NEB, Massachusetts, USA) for the reactions and *Actin2* as the internal reference. Specific Primer 5.0 software was used to design the primers for the experiment (Table 7). Three technical replicates and three biological replicates were analyzed.

### 4.7. Parallel Reaction Monitoring (PRM) Analysis

We validated the iTRAQ data using LC-PRM/MS (Shanghai Applied Protein Technology, Shanghai, China) [68]. In brief, we followed the instructions of iTRAQ for peptide preparation, 10 fmol heavy isotope-labeled peptide fragment DSPSAPVNVTVR (framed V: a heavy isotope label synthesized by GL Biochem) was added into each sample as the internal standard. An Easy nLC-1200 system (Thermo Scientific, MA, USA) was used for separating the tryptic peptides. The 1 h liquid chromatography had acetonitrile gradients ranging from 5% to 35% over 45 min. The Q Exactive Plus mass spectrometer (Thermo Scientific) was used in the positive ion mode for a full MS1 scan with a resolution of 60,000 at 200 m/z, an automatic gain control (AGC) target value of 3.0 × 10^−6^, and a 200 ms maximum ion injection time. After the full MS scans, 20 PRM scans were run with the same parameters, except for the resolution and maximum ion injection time, which were adjusted to 30,000 and 120 ms, respectively. A 1.6 Th window was set for isolating the target peptides. Normalized collision energy of 27 eV in a higher-energy dissociation (HCD) collision cell was set for ion activation/dissociation. Skyline was used for raw data analysis to quantify the signal intensity of each individual peptide sequence whose abundance changed significantly. After the quantification, the data were normalized to the standard reference. Each sample had three biological replicates. 

### 4.8. Flavonoid and Anthocyanin Measurement

To measure flavonoid contents, 0.1 g of 12-day-old seedlings exposed to UV-B were quantified according to Kucera [69]. The measurement of anthocyanin levels for 0.1 g of 12-day-old seedlings exposed to UV-B had been described previously [70,71].

### 4.9. Photochemical Activity Measurement

We measured the chlorophyll fluorescence parameters, including the maximal PSII efficiency (*Fv/Fm*) and non-photochemical quenching (NPQ), using Chlorophyll Fluorescence Imager (Technologica, Colchester, UK). The samples were put in the dark for 20 min before measurements [72]. 

### 4.10. Measurements for IAA, ABA and JA

We measured the IAA, ABA, and JA content in each sample. Two hundred milligrams of each liquid nitrogen-frozen fresh sample was homogenized in the TissueLyser (QIAGEN, Dusseldorf, Germany), then send to Hunan Provincial Key Laboratory of Phytohormones (Hunan Agricultural University, Changsha, China) for further extract and measurements according to Zhou [73]. Each sample had three biological replicates.

### 4.11. Data Analysis

GraphPad Prism 7.0 software was used for statistical analysis and drawing. The statistical analysis includes One-Way ANOVA analysis, Dunnett’s multiple tests and difference significance analysis. Differences were considered significant for *P* < 0.05. Adobe Photoshop CC was used for image processing, and Adobe Illustrator CC was used for chart layout.

## 5. Conclusions

**Figure 12:**
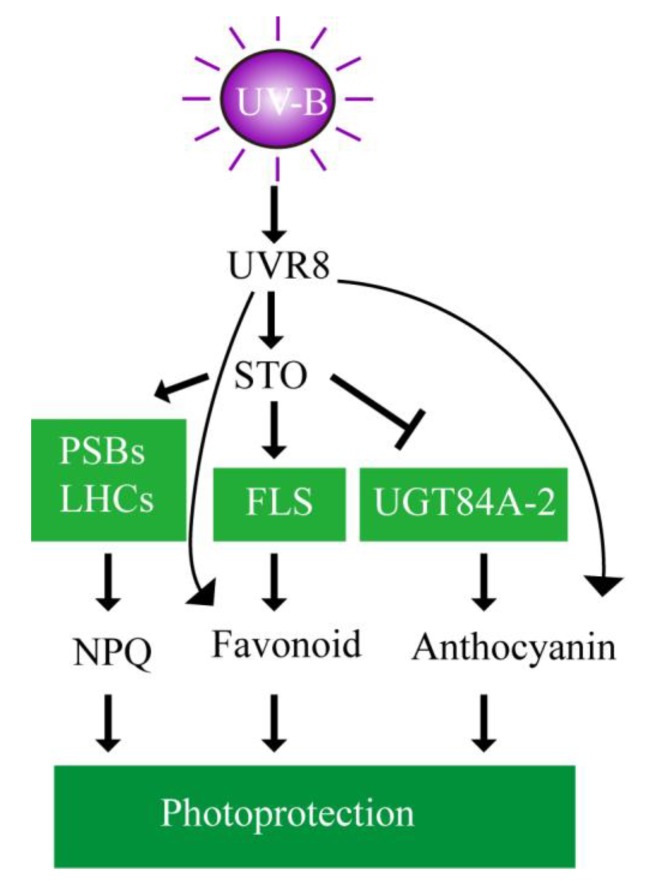
Schematic model of photoprotection mediated by STO in the presence of UV-B radiation. The putative mechanisms are schemed to depict the involvement of STO in UV-B signaling. UV-B is received by the photoreceptor UVR8 to induce STO accumulation in plants. STO protects the photosynthetic machinery by stabilizing the antenna system and releasing excess energy. Meanwhile, STO represses anthocyanin synthesis and promotes flavonoid synthesis for photoprotection.

The putative mechanism is schemed (Figure 12) to depict the involvement of STO in UV-B signaling. UV-B is received by the photoreceptor UVR8 to induce STO accumulation in plants. STO promotes the accumulation the PSBs and LHCs to stabilize the antenna system and release excess energy for protecting the photosynthetic machinery. Meanwhile, STO represses the accumulation of UGT84A2 to decrease anthocyanin synthesis and promotes the accumulation of FLS to increase flavonoid synthesis for photoprotection. UV-B increases flavonoid and anthocyanin synthesis, but does not depend on STO.

## Figures and Tables

**Table 1:** Information about the proteins

Database	NO.	Total Spectra	Peptide Spectrum Match	Peptides	Unique Peptides	Protein Groups
TAIR	1	312,550	94,209	41,480	36,822	7306
TAIR	2	307,230	94,057	40,076	35,436	7130
TAIR	3	306,008	93,046	38,283	33,967	6931
TAIR	Total	925,788	281,312	54,992	48,791	8212

**Table 2:** Number of differentially expressed proteins in each comparison group.

Comparison Group	Up-Regulated	Down-Regulated	In Total
(+)WT/(-)WT	27	14	41
(+)*sto*/(-)*sto*	213	109	322
(+)*sto*/(+)WT	292	322	614
(-)*sto*/(-)WT	45	39	84

Note: Fold change more than 1.2 times is up-regulated, and less than 0.83 times is down-regulated, *P* < 0.05.

**Table 3:** Fold changes of flavonoid pathway proteins in different groups.

Gene ID	Description	(+)WT/ (-)WT	(+)*sto*/ (-)*sto*	(-)*sto*/ (-)WT	(+)*sto*/ (+)WT
*AT2G37040.1*	PAL1, phenylalanine ammonia-lyase 1	1.6	1.31	1.13(NS)	0.92
*AT3G53260.1*	PAL2, phenylalanine ammonia-lyase 2	1.35	1.18	0.99(NS)	0.87
*AT5G13930.1*	CHS, chalcone and stilbene synthase family protein	1.86	1.39	1.23	0.92
*AT5G08640.1*	Flavonol synthase 1	1.32	1.12	1.07(NS)	0.9
*AT3G21560.1*	UDP-Glycosyltransferase 84A2	1.57	1.35	1.04(NS)	0.89
*AT1G43620.1*	UDP-Glycosyltransferase superfamily protein	1.22	1.05(NS)	0.96(NS)	0.82

Note: Fold change more than 1.2 times is up-regulated, and less than 0.83 times is down-regulated, *P* < 0.05. ‘NS’ means no significance.

**Table 4:** Fold changes of differential proteins in the photosynthesis pathway in different groups.

Gene ID	Description	(+)WT/ (-)WT	(+) *sto*/ (-) *sto*	(-)*sto*/ (-)WT	(+)*sto*/ (+)WT
*AT2G05620.1*	PGR5, response to water deprivation, photosynthetic electron transport in PSI	1.10(NS)	0.90(NS)	0.95(NS)	0.78
*AT3G54890.4*	Chlorophyll binding component of the light harvesting complex associated with PSI	1.08(NS)	0.93(NS)	0.90(NS)	0.77
*AT1G31330.1*	PSAF, PSI subunit F	1.11(NS)	1.00(NS)	0.91(NS)	0.81
*ATCG01010.1*	NADH dehydrogenase activity	1.12(NS)	0.92(NS)	0.95(NS)	0.78
*AT5G58260.1*	Oxidoreductases, acting on NADH or NADPH, quinone or similar compound as acceptor	1.07(NS)	0.96(NS)	0.92(NS)	0.82
*AT3G01440.1*	Oxygen evolving enhancer 3 (PsbQ) family protein	1.05(NS)	0.97(NS)	0.9	0.83
*ATCG00710.1*	A component of the photosystem II oxygen evolving core	1.19	0.94(NS)	1.04(NS)	0.82
*AT2G40100.1*	LHCB4.3 (light harvesting complex PSc), chlorophyll binding	1.03(NS)	0.85	0.97(NS)	0.8
*AT1G15820.1*	LHCB6, PSII antenna complex	1.09(NS)	0.96(NS)	0.92(NS)	0.81
*AT1G61520.2*	LHCA3, chlorophyll binding	1.13(NS)	0.93(NS)	1.00(NS)	0.83
*AT2G05070.1*	LHCB2.2, constitute the antenna system of the photosynthetic apparatus, chlorophyll binding	1.08(NS)	0.98(NS)	0.91(NS)	0.83
*AT1G19150.1*	LHCA6, chlorophyll binding PSI type II chlorophyll a/b-binding	1.11(NS)	0.94(NS)	0.89(NS)	0.76
*AT2G34430.1*	LHB1B1, chlorophyll binding PSII type I chlorophyll a/b-binding	1.07(NS)	0.91(NS)	0.79	0.67
*AT2G34420.1*	LHB1B2, chlorophyll binding	0.94(NS)	0.88	0.89	0.83
*AT2G20890.1*	PSB29, controls the assembly of the PSII complex	1.14(NS)	0.99(NS)	0.91(NS)	0.79

Note: Fold change more than 1.2 times is up-regulated, and less than 0.83 times is down-regulated, *P* < 0.05. ‘NS’ means no significance.

**Table 5:** Fold changes of hormone-related proteins.

Gene ID	Description	(+)WT/ (-)WT	(+)*sto*/ (-)*sto*	(-)*sto*/ (-)WT	(+)*sto*/ (+)WT
*AT2G41430.1*	ERD15, an attenuator of plant ABA, response to light intensity, water deprivation, cold tolerance, salt stress	0.78	1.62	0.92(NS)	1.91
*AT2G39800.1*	P5CS1, response to ABA, desiccation, oxidative stress, salt stress, water deprivation, root development	1.00(NS)	1.09(NS)	0.75	0.82
*AT5G25610.1*	RD22, response to abscisic acid, response to desiccation, salt stress	0.97(NS)	0.91(NS)	0.8	0.75
*AT5G59320.1*	LTP3, involved in lipid transport, response to ABA, water deprivation	1.19(NS)	0.99(NS)	0.78(NS)	0.69
*AT5G01990.1*	Auxin efflux carrier family protein	0.94(NS)	1.79	0.86(NS)	1.64
*AT1G54990.1*	AXR4/RGR1, auxin-resistant root growth	1.04(NS)	1.4	1.04	1.41
*AT2G33830.1*	DRM2, response to stress and environmental factors	0.76	0.86	1.11	1.26
*AT3G45140.1*	LOX2, response to water deprivation, JA, oxidation-reduction process, lipid oxidation	1.03(NS)	1.02(NS)	0.79	0.78
*AT1G62380.1*	ACO2, response to fatty acid, defense response, ethylene stimulus, ethylene biosynthetic process, oxidation-reduction process, cytokinin, salt stress	0.97(NS)	0.93(NS)	1.4	1.34

Note: Fold change more than 1.2 times is up-regulated, and less than 0.83 times is down-regulated, *P* < 0.05. ‘NS’ means no significance.

**Table 6:** Representative protein quantitative confirmation with PRM analysis.

Accession Number	Gene Symbol	Signature Peptides	PRM				iTRAQ			
			A	B	C	D	A	B	C	D
*AT2G37040.1*	PAL1	LAGISSGFFDLQPK	2.89	1.95	1.60	1.14	1.60	1.3	1.13	0.92
		FLNAGIFGSTK								
		EELGTELLTGEK								
*AT3G21560.1*	UGT84A2	YDFFDDGLPEDDEASR	2.59	2.59	1.14	1.30	1.57	1.35	1.04	0.89
		IVEWCSQEK								
*AT1G15820.1*	LHCB6	WVDFFNPDSQSVEWATPWSK	1.16	1.07	1.00	0.79	1.09	0.96	0.92	0.81
		DGVYEPDFEK								
		SWIPAVK								
*AT1G03130.1*	PSAD-2	AQVEEFYVITWNSPK	1.31	0.66	1.14	0.52	1.10	0.94	0.91	0.78
		ITYQFYR								
		EGPNLLK								

Note: The letter A means (+)WT/(-)WT, B means (+)*sto*/(-)*sto*, C means (-)*sto*/(-)WT, D means (+)*sto*/(+)WT.

**Table 7:** The primers of qRT-PCR.

Name	Primer Name	Sequence
*Actin*	ACTIN2-F	GTTGGGATGAACCAGAAGGA
	ACTIN2-R	GCTCTTCAGGAGCAATACGAAG
*PAL1*	PAL1-QF	GATTATGGATTCAAGGGAG
	PAL1-QR	TTTGCGAGACGAGATTAG
*FLS*	FLS-QF	ATACAGGGAGGTGAATGAA
	FLS-QR	ACACGGCGGATAATAGTT
*CHS*	CHS-QF	ACGTCACGTGTTGAGCGAGTATGG
	CHS-QR	GAGGAACGCTGTGCAAGACGACTG
*LHCA3*	LHCA3-QF	TTCTTCACTTACCTCCTCTG
	LHCA3-QR	GTCTGTTGGCTCCTTGCT
*LHCA2*	LHCA2-QF	TAGCCTCCCTGGTGACTT
	LHCA2-QR	GGATTCCGATCTTCGTTAG
*PSAD-2*	PSAD-2-QF	GCCATAACAACCACTACTTC
	PSAD-2-QR	ACTGGAGCTTCTTTCACG
*DREB2A*	DREB2A-QF	TGAAAGGTAAAGGAGGAC
	DREB2A-QR	CCAAAGGACCATACATAGC
*DREB1A*	DREB1A-QF	GTGGGTTTGTGAGGTTAGAG
	DREB1A-QR	CCTTAGCGCAAGTTGATT
*RD22*	RD22-QF	CCAAACACTCCCATTCCC
	RD22-QR	ACACCTCCCTTTCCAACG
*ABI5*	ABI5-QF	GGTGAGAATCATCCGTTTA
	ABI5-QR	TCCTCTGCGTTCCAAATA
*P5CS1*	P5CS1-QF	AGGGAAAGTTCCAGAAAG
	P5CS1-QR	CATAACTAAGCGAGCCAC
*SAD2*	SAD2-QF	CTTATGACCGACAGAAACA
	SAD2-QR	CAACAGTGAGACGCAGAT

## Abbreviations

STO	Salt Tolerance
HY5	Elongated Hypocotyl 5
iTRAQ	Isobaric Tags for Relative and Absolute Quantification
PRM	Parallel Reaction Monitoring
GO	Gene Ontology
KEGG	Kyoto Encyclopedia of Genes and Genomes
UVR8	UV Resistance Locus 8
COP1	Constitutively Photomorphogenic 1
RUPs	Repressor of UV-B Photomorphogenesis
PIF4	Phytochrome Interacting Factor 4
PAL	Phenylalanine Ammonia Lyase
CHS	Chalcone Synthase
DFR	Dihydroflavonol Reductase
FLS	Flavonol Synthase
